# Evaluating the seasonal efficacy of commonly used chemical treatments on *Varroa destructor* (Mesostigmata: Varroidae) population resurgence in honey bee colonies

**DOI:** 10.1093/jisesa/ieae011

**Published:** 2024-05-28

**Authors:** Cameron J Jack, Humberto Boncristiani, Cody Prouty, Daniel R Schmehl, James D Ellis

**Affiliations:** Entomology and Nematology Department, University of Florida, Gainesville, FL, USA; Entomology and Nematology Department, University of Florida, Gainesville, FL, USA; Inside The Hive Media & Consulting Inc., Odenton, MD, USA; Entomology and Nematology Department, University of Florida, Gainesville, FL, USA; Department of Entomology, University of Minnesota, St. Paul, MN, USA; Entomology and Nematology Department, University of Florida, Gainesville, FL, USA; Entomology and Nematology Department, University of Florida, Gainesville, FL, USA

**Keywords:** *Varroa destructor*, resistance, season, amitraz, oxalic acid

## Abstract

The purpose of this research was to determine how common chemical treatments influence *Varroa destructor* (Anderson and Trueman) population resurgence rates (defined as time posttreatment for mite populations to reach 3 mites/100 adult bees) in managed honey bee (*Apis mellifera* L.) colonies seasonally. We conducted 2 experiments that followed the same basic protocol to address this purpose. We established 6 treatment groups in Experiment 1 in the fall of 2014: untreated control, Apivar, Apistan, CheckMite+, ApiLifeVar, and Mite Away II applied to 10 colonies per treatment. In Experiment 2, we applied 8 chemical treatments to each of 4 seasonal (spring, summer, fall, and winter) cohorts of honey bee colonies to determine how mite populations are influenced by the treatments. The treatments/formulations tested were Apivar, Apistan, Apiguard, MAQS, CheckMite+, oxalic acid (dribble), oxalic acid (shop towels), and amitraz (shop towels soaked in Bovitraz). In Experiment 1, Apivar and Mite Away II were able to delay *V. destructor* resurgence for 2 and 6 months, respectively. In Experiment 2, Apiguard, MAQS, oxalic acid (dribble), and Bovitraz treatments were effective at delaying *V. destructor* resurgence for at least 2 months during winter and spring. Only the Bovitraz and MAQS treatments were effective at controlling *V. destructor* in the summer and fall. Of the 2 amitraz-based treatments, the off-label Bovitraz treatment was the only treatment to reduce *V. destructor* populations in every season. The data gathered through this study allow for the refinement of treatment recommendations for *V. destructor*, especially regarding the seasonal efficacy of each miticide and the temporal efficacy posttreatment.

## Introduction

Beekeepers worldwide are facing devastating losses caused by *Varroa destructor* in their managed honey bee (*Apis mellifera*) colonies (Boncristiani et al. 2021, Insolia et al. 2022, Stahlmann‑Brown et al. 2022, Albarrak and Gray 2023, Bruckner et al. 2023, Gray et al. 2023). For most commercial beekeepers, *V. destructor* control is achieved by treating colonies that exhibit high mite levels with synthetic miticides (Haber et al. 2019). However, *V. destructor* has evolved resistance to several of these chemicals over the past few decades (Mitton et al. 2022). Beekeepers also use other nonsynthetic chemicals and mechanical manipulations to control *V. destructor*, but these are rarely sufficient as stand-alone treatments (Jack and Ellis 2021). The lack of effective *V. destructor* controls has left commercial beekeepers with few options other than continuing to use the same chemical treatments year after year (Underwood et al. 2019), thus exacerbating the development of *V. destructor* resistance to commercial products.

One strategy to improve beekeepers’ chances of controlling *V. destructor* populations is to improve the timing of their treatments. If beekeepers do not treat during the most appropriate time, the treatment likely will be ineffective and may even possibly harm the bees (Delaplane and Hood 1997). This wastes the beekeepers’ resources and continues the trend of insufficient *V. destructor* control. Treatments must also be timed to match the natural biology of honey bees and the mites. For example, *V. destructor* population growth within a honey bee colony is dependent upon the colony’s brood rearing activity because the mites can only reproduce in cells of developing bees (Wilkinson and Smith 2002, Rosenkranz et al. 2010). *Varroa destructor* populations change seasonally, following the availability of brood (Jack et al. 2023). Given *V. destructor* spend most of their lives inside honey bee brood cells (Rosenkranz et al. 2010), they are protected from most chemical treatments that are not able to penetrate the wax capping. Therefore, the efficacies of some treatments are maximized when less brood is present in the nest. Armed with this knowledge, beekeepers using a given *V. destructor* control must consider applying the treatment at a time when it will be most effective (Delaplane and Hood 1997, Currie and Gatien 2006).

While miticide efficacies vary, the use of 1 product for an extended time will be selected for resistance in the mite. Selection pressures for resistance are greatest when a product has an efficacy of > 90% (Milani 1999). Therefore, it is necessary to develop treatment regimens based upon a rotation of miticides that have different modes of action, even if these treatments control *V. destructor* at lower efficacies (Sammataro et al. 2005, Roth et al. 2020, Jack and Ellis 2021). Of the synthetic chemical treatments used to control *V. destructor* in the United States [Apistan (*tau*-fluvalinate), CheckMite+ (coumaphos), and Apivar (amitraz)], beekeepers use Apivar most, as mite resistance to the other 2 treatments is well documented (Elzen et al. 1999, Milani 1999, Elzen and Westervelt 2002, Pettis 2004, Maggi et al. 2009, 2010, González‑Cabrera et al. 2013). Unfortunately, reports of mite resistance to amitraz have become more common since 2016 (Kamler et al. 2016, Rinkevich 2020). Thus, US beekeepers still need to utilize products such as Apiguard (thymol), MAQS (formic acid), and/or Api-Bioxal (oxalic acid) even though efficacies are typically lower for these treatments than they are for the synthetic chemical treatments (Jack and Ellis 2021).

Another strategy to combat *V. destructor* is to search for new active ingredients that are toxic to the mite but are relatively safe for honey bees (Bahreini et al. 2020, Jack et al. 2022). However, this process is time-consuming and expensive, and it can often take a decade or longer for a company to create and register a product to be used by beekeepers. Thus, many US beekeepers create their own low-cost treatments using active ingredients found in products not registered for use in honey bee colonies. One common beekeeper-created treatment involves using products registered for the control of cattle pests, products such as Taktic (Chen et al. 2007, Oliver 2014) or Bovitraz (Honey Bee Health Coalition 2021). Beekeepers formulate these treatments at higher concentrations of amitraz than what is in the commercially available product Apivar. Beekeepers may use other off-label products as well. For example, some beekeepers mix oxalic acid (OA) into glycerin in an effort to extend OA’s efficacy for longer periods (Oliver 2017). Beekeepers typically apply these off-label treatments by soaking absorbent industrial wiping cloths (shop towels) in mixtures of the compounds and placing the towels on the top bars of frames above the brood area of the hive. These treatments have been used by many beekeepers around the United States for years, though we discourage the off-label use of a product due to concerns for human and environmental health.

While there have been investigations into the most suitable timing of miticide applications for *V. destructor* control (Strange and Sheppard 2001), the length of time it takes after miticide applications for *V. destructor* to reach the economic threshold of 3 mites/100 adult bees (Jack and Ellis 2021), which we define as population resurgence, has not been studied. The overall goal of the research presented herein was to determine how commonly used chemical treatments influence *V. destructor* population resurgence seasonally so that specific recommendations on product efficacy can be made to commercial beekeepers. We conducted 2 research experiments to address this goal. The results of both experiments allow us to revise the best management recommendations for *V. destructor* control based on the seasonal efficacies of the tested treatments.

## Materials and Methods

### Experiment 1

In the fall of 2014, 60 colonies of European-derived honey bee stock placed in 3 locations within a 30 km radius of the University of Florida Bee Biology Unit (29.62°N, −82.36°W) were used to determine *V. destructor* population resurgence. Forty of these experimental honey bee colonies located at an apiary in Micanopy, FL, USA, received 1 of 4 treatments (10 colonies per treatment): (i) Apiguard (thymol), (ii) Apistan (*tau*-fluvalinate), (iii) Apivar (amitraz), and (iv) CheckMite+ (coumaphos). The treatments were administered according to the product labels. At the start of the trial, all the colonies in the Micanopy apiary (treatment groups 1–4) were equalized to ensure that they were of similar size (number of bees; area of brood), strength (honey and pollen stores; absence of disease), and *V. destructor* infestation before beginning the trial due to their co-location in the same apiary. Additionally, 15 colonies were located at the Bee Biology Unit, with 1 set of 10 colonies treated with Mite Away II (formic acid) and 1 set of 5 colonies designated as controls (no miticide treatment). One additional set of 5 untreated control colonies was located at an apiary in High Springs, FL, USA. The colonies in the Bee Biology Unit and the High Spring apiary were not equalized as the Micanopy colonies. As colonies available for this study were limited, these colonies at the Bee Biology Unit and High Springs apiaries were not intended to be compared with other treatments. The control colonies were only intended to validate *V. destructor* population growth in a colony when treatment was not used. In other words, the intention was that the comparisons on mite populations were intended to be made within treatment (i.e., change relative to starting infestation) rather than between treatments.

*Varroa destructor* populations were measured by collecting a subsample of ~300 adult honey bees from 1 frame from the center of the brood box of each colony. The honey bees were chilled in a cooler and transported to the laboratory, where mite populations for each colony were recorded by performing soapy water washes using a double-sieve as described by Dietemann et al. (2013). Mite infestation rates described as # mites/100 adult bees were calculated for each colony. The sampling events occurred at a series of 6-time points for each colony to characterize miticide efficacy and *V. destructor* population resurgence after treatment. The sampling time points included: (i) project initiation (pretreatment *V. destructor* numbers), (ii) end of treatment (when miticide product is removed), (iii) 1-month posttreatment, (iv) 2 months posttreatment, (v) 4 months posttreatment, and (vi) 6 months posttreatment. Mite populations in the control colonies were measured at project initiation, at the end of the treatment period (when the other miticides were removed), and each month for 7 months. With these data, we were able to calculate miticide efficacy and *V. destructor* population resurgence.

### Experiment 2

The seasonality miticide study was conducted over the course of 4 seasons, spanning October 2020 to January 2022. The fall season began in October 2020. Winter began in January 2021. Spring began in May 2021, and summer began in August 2021. All the colonies used in this study (*N* = 412) were of European-derived honey bee stock and were maintained at the University of Florida’s Beef Research Unit (UFBRU) in Gainesville, Florida, USA (29.74°N, 82.26°W). We tested the efficacy of 8 different chemical treatments in reducing *V. destructor* populations within each season. All commercial products were applied according to their respective labels appropriate to the size and strength of the colonies. All other treatments were applied according to common beekeeper use patterns (described in *Treatment Application* section below). The chemical treatments/formulations tested were Apivar (amitraz), Apistan (*tau*-fluvalinate), Apiguard (thymol), MAQS (formic acid), CheckMite+ (coumaphos), OA (dribble), OA (shop towels), and amitraz (shop towels soaked in Bovitraz). A separate research study through which the investigators measured the effect of seasonal growth of *V. destructor* populations in untreated colonies (*N* = 160) was conducted in the same region during the same period as the present research (Jack et al. 2023). This research was intended to provide a baseline of *V. destructor* population growth, to which the research presented herein can be compared. Thus, there were no colonies designated as controls used in the experiment described here.

During the project, all colonies were managed in wooden 10-frame deep Langstroth hive bodies that included a migratory lid and external feeder jar. The hives were placed on modified 4-way wooden pallets commonly used by commercial beekeepers in the United States. All colonies were managed according to standard management practices for the region and the season (swarm control, supplementary feeding, small hive beetle trapping, etc.). The colonies were equalized to ensure that they were of similar size (number of bees; area of brood) and strength (honey and pollen stores; signs of disease) before beginning each seasonal trial. After equalization, no brood, bees, or resources were shared between the colonies to prevent the mechanical transfer of *V. destructor* by the researchers. Prior to the start of each trial, colonies were randomly assigned to 1 of the 8 treatment groups using a random number generator. Each treatment group was separated by a minimum of 50 m to minimize the amount of drifting between colonies of the various treatment groups.

### Data Collection

Prior to the start of each seasonal trial, the baseline *V. destructor* infestation level was determined for each colony using alcohol washes to determine # mites/100 adult honey bees following the methods described by Dietemann et al. (2013). After the miticide treatment had been applied, the experimental colonies were sampled monthly until the average *V. destructor* infestation level of an individual treatment group reached the economic threshold of 3 mites/100 adult honey bees, at which time a treatment group was removed from the study. A seasonal study concluded that all remaining treatment groups averaged *V. destructor* infestation levels that reached or exceeded the economic threshold. The lone exception to this concerns the fall trial, in which all treatment groups ceased to be monitored after 3 months. We also recorded colony mortality during each seasonal trial.

### Treatment Application

All commercially available treatments were applied as directed by the label. These treatments included Apivar (3.3.% amitraz), Apistan (10.25% *tau*-fluvalinate), Apiguard (25% thymol), MAQS (46.7% formic acid), CheckMite+ (10% coumaphos), OA dribble (2.9% OA solution dribbled once per week for 3 wk). The Apivar, Apistan, and CheckMite+ treated colonies all received 2 strips for a 42-day period. The Apiguard treatment was applied in ready-to-use aluminum trays. The foil lid was completely pulled back, and the tray was left in the hive for 2 wk before it was removed and replaced with another tray applied in the same manner. MAQS was applied using the 7-day treatment option, where 2 strips were laid across the top bars and were removed after 14 days.

The 2 remaining chemical treatments were prepared and applied according to beekeeper recommendations. The OA (shop towel) treatment was prepared following the protocol outlined in Oliver (2017). The protocol for creating 10 shop towels (Scott) soaked in OA is as follows: (i) Mix 120 g of OA with 100 mL of warm water. (ii) Add 130 mL of glycerol to the OA solution. (iii) Pour the solution into a sealable container. (iv) Add 10 shop towels to the container to absorb the solution. (v) Place 1 shop towel on top of the frames to treat each hive. For the off-label use of Bovitraz (12.5% amitraz), we followed the protocol that is most common among commercial beekeepers. The protocol for creating 10 shop towels soaked Bovitraz is as follows: (i) mix 100 mL of Bovitraz with 100 mL of canola oil, (ii) pour the solution into a sealable container, (iii) add 10 shop towels to the container to absorb the solution, and (iv) place 1 shop towel (now 6.25% amitraz) on top of the frames to treat each hive.

Within a seasonal trial, every colony was assigned to 1 of the 8 treatments. Once a seasonal trial was completed, the surviving colonies were then equalized and randomized to await their participation in the next trial period. Not all colonies were included in each seasonal trial, but they were used as necessary to ensure that at least 10–16 colonies could be tested for each treatment every season.

### Statistical Analysis

Analyses were the same for both experiments, though only 1 season was represented in Experiment 1. Comparisons could be made across seasons in Experiment 2. In Experiment 1, the untreated controls were not included in the analyses (given that they were only to serve as a point of reference), and Mite Away II was removed since this treatment, along with the controls, was applied at a different location with possible differences in environmental conditions. Colony survival was analyzed using mixed effects Cox proportional hazards models in the package coxme (Therneau 2015). In Experiment 1, survival was analyzed to the end of the experiment (month 7). In Experiment 2, we analyzed survival during the treatment period at 3 months, given the fact that the fall season only lasted 3 months, and we were trying to match all trials for this parameter. Colonies that survived more than 3 months were coded as having survived 3 months (right censored survival analysis). The difference in *V. destructor* infestation rates was calculated as the number of mites per 100 bees from the 1st sample (pretreatment) subtracted from the 2nd sample (Experiment 1) and 3rd sample (Experiment 2), which represents the immediate posttreatment sample. Due to differences in sampling technique between experiments, the sample number is different, but the biological significance is equivalent. These 2 times were selected for comparison because each treatment in every season had at least 3 periods of data collection. Furthermore, all treatments were fully applied within this period. The differences in *V. destructor* infestation rates were analyzed using a linear model, with the model structure for Experiment 1: [Difference in *V. destructor* infestation ~ miticide × initial *V. destructor* infestation], and Experiment 2: [Difference in *V. destructor* infestation ~ miticide × season + initial *V. destructor* infestation]. In Experiment 2, the initial *V. destructor* infestation was originally involved in a 3-way interaction, but given that this variable was not significantly involved in any 2- or 3-way interactions with other terms, the 3-way interaction was removed for simplicity. Following the overall analysis, we compared miticides across seasons and miticides within seasons. Each miticide was then subset by season.

## Results

### Experiment 1

There was no significant effect of any treatment on colony survival to month 7 (*χ*^2^_3,40_ = 2.58, *P* = 0.462, Supplementary Fig. S1).

**Fig. 1.**
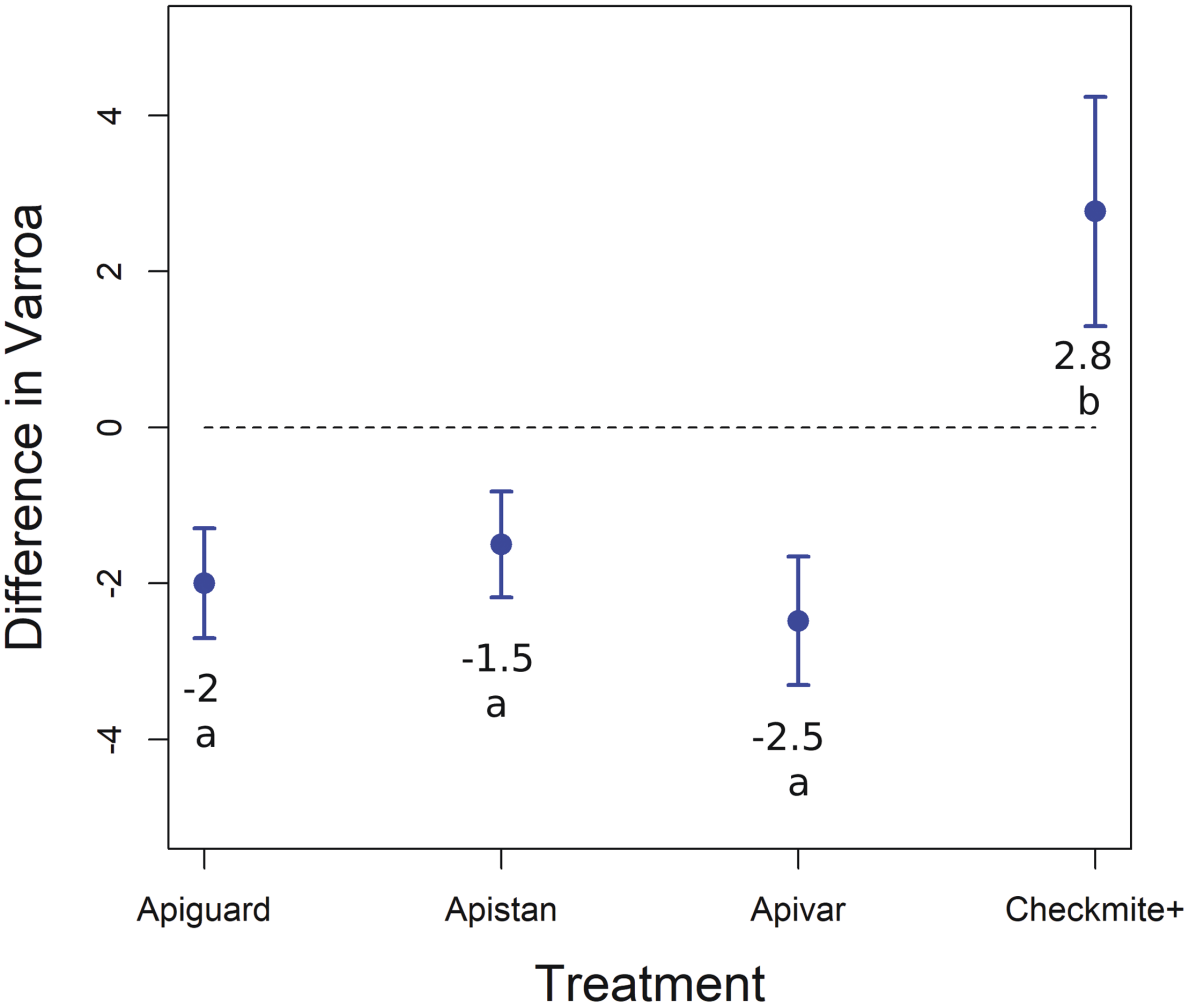
Experiment 1—The difference between the average number of *Varroa destructor* per 100 bees in colonies immediately after treatment (October 2014) and pretreatment (September 2014) in honey bee colonies treated with a miticide. Negative values indicate that the *V. destructor* populations decreased, while positive values indicate the populations increased during the treatment application period.

*Varroa destructor* population resurgence in colonies after treatment with a miticide is shown in Table 1, and the relative efficacy of the 5 miticides tested in Experiment 1 can be viewed in Fig. 1 and Supplementary Table S1. There was a significant effect of treatment on the difference in *V. destructor* infestation rates (mites/100 adult bees) pretreatment and immediate posttreatment (*F*_3,32_ = 12.87, *p* < 0.001). Post hoc comparisons show that mite populations increased the most for colonies treated with CheckMite+ and less so for colonies treated with Apiguard, Apistan, and Apivar. Both the Mite Away II and the controls were included in Table 1 for completeness of information, as no comparisons were made across treatments in this table.

**Table 1.**
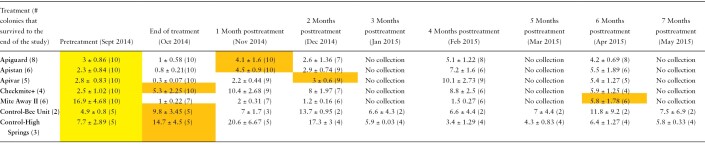
Experiment 1. The growth of *Varroa destructor* populations in honey bee colonies after treatment with a miticide. The data are an average number of *V. destructor* per 100 adult bees (number of colonies sampled), or *V. destructor* infestation rates. The data in the yellow-highlighted column represent the infestation rates in colonies *prior to treatment* with a miticide. The data in the orange-highlighted boxes indicate when the infestation rates exceeded 3 mites per 100 adult bees. This level is an economic threshold that warrants beekeeper use of chemical intervention to reduce *V. destructor* populations. Formic acid-treated colonies received their treatment and were sampled for *V. destructor* 3 wk later than the other colonies in the study. This happened during each sampling period for the colonies treated with formic acid

There was no significant effect of the pretreatment mite infestation rates on the difference in *V. destructor* infestation rates from pretreatment to immediately posttreatment (*F*_1,32_ = 3.79, *P* = 0.06), but there was a significant interaction between pretreatment mite infestation rates and the difference in *V. destructor* infestation rates from pretreatment to immediately posttreatment (*F*_3,32_ = 13.73, *P* < 0.001). This means that when averaging across all treatments, starting *V. destructor* populations did not influence how well a treatment would reduce a mite population (Supplementary Fig. S2A). However, treatments were significantly different regarding the directionality of the effect of the pretreatment on the difference in *V. destructor* infestation rates (Supplementary Fig. S2B).

### Experiment 2—Survival During the Treatment Period

**Fig. 2.**
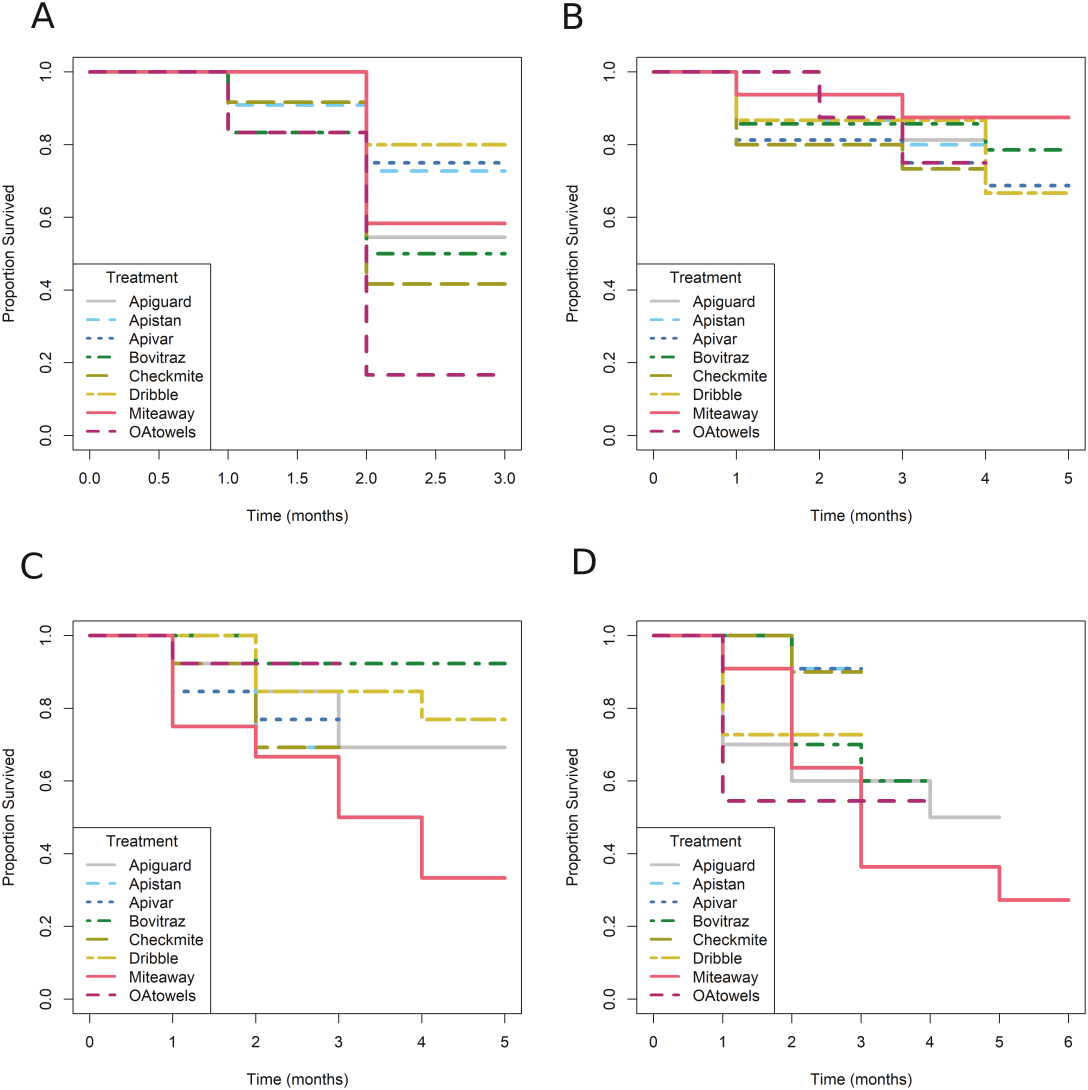
Experiment 2—Proportion of experimental honey bee colonies that survived monthly in each of the 4 seasonal trials: A) Fall, B) Winter, C) Spring, and D) Summer. OA, oxalic acid.

Overall, there were no significant impacts of treatment (*χ*^2^_7,33_ = 4.68, *P* = 0.699), season (*χ*^2^_3,33_ = 1.76, *P* = 0.624), or the interaction between the 2 (*χ*^2^_21,33_ = 10.88, *P* = 0.965) on the proportion of colonies that survived 2 months posttreatment (Fig. 2).

### Experiment 2—Interactions

Overall, treatment and season interacted significantly to affect the difference in mite infestation rates from the 1st to the 3rd sampling period (*F*_21, 235_ = 4.5, *P* < 0.001). Thus, the impact of treatment on the difference in mite infestation rates was analyzed separately by season. Treatment application affected these differences significantly each season: fall (*F*_7,30_ = 3.33, *P* = 0.01), winter (*F*_7,77_ = 2.77, *P *= 0.013), spring (*F*_7,72_ = 5.59, *P *< 0.001), and summer (*F*_7,53_ = 3.67, *P *= 0.003). Multiple comparisons of these effects are shown in Table 2 and discussed in *Experiment 2—Seasonal Efficacy* section.

**Table 2.** Experiment 2. The difference in *Varroa destructor* infestation rates between pretreatment and 2 months posttreatment (3rd sample event) for each season. The data are mean ± s.e. (percentage change in *V. destructor* infestation rates between pretreatment and 2 months posttreatment ± s.e.) and *N* = surviving colonies at the time of the 3rd sample event. Negative efficacies represent a decrease in *V. destructor* infestation rates (i.e., the treatment reduced infestation rates). Positive efficacies mean the infestation rates increased over the treatment period. Comparisons are made within seasons across treatments, i.e., vertical means with different letters are significantly different *α* ≤ 0.05. OA, oxalic acid

Treatment	Fall (October–December)	Winter (January–May)	Spring (May–September)	Summer (August–January)
Apiguard	1 ± 0.69 ab (37.97 ± 21.75) *N* = 3	−0.94 ± 0.55 a (−76.19 ± 11.62) *N* = 14	−6.4 ± 1.61 a (−95.48 ± 2.73) *N* = 11	−3.76 ± 1.77 ab (−6.68 ± 88.39) *N* = 6
Apistan	20.68 ± 8.71 b (924.25 ± 647.69) *N* = 6	−1.44 ± 0.39 ab (−36.63 ± 21.05) *N* = 12	−1.14 ± 2.64 b (134.45 ± 99.41) *N* = 9	2.04 ± 1.27 ab (226.76 ± 139.54) *N* = 10
Apivar	1.27 ± 1.24 ab (33.04 ± 30.14) *N* = 9	−1.5 ± 1.22 ab (−34.57 ± 38.41) *N* = 11	0.57 ± 1.49 ab (7.93 ± 28.28) *N* = 10	1.53 ± 1.45 ab (66.15 ± 35.39) *N* = 10
Bovitraz	−1.76 ± 0.75 a (−39.49 ± 14.06) *N* = 6	−1.16 ± 0.37 ab (−65.08 ± 16.37) *N* = 6	−8.36 ± 2.69 a (−94.67 ± 3.08) *N* = 11	−3.15 ± 2.18 ab −14.79 ± 35.01 *N* = 7
CheckMite+	10.43 ± 6.18 ab (1114.4 ± 667.03) *N* = 3	−0.46 ± 0.7 ab (10 ± 20.7) *N* = 12	0.46 ± 2.58 b (74.41 ± 76.59) *N* = 9	4.72 ± 2.02 b (171.38 ± 53.3) *N* = 9
MAQS	−2.58 ± 2.56 ab (63.89 ± 97.26) *N* = 4	−1.36 ± 0.25 a (−86.3 ± 9.01) *N* = 11	−8.72 ± 2.33 a (−92.37 ± 6.34) *N* = 8	−2.84 ± 1.79 a (−83.71 ± 7.54) *N* = 6
OA Dribble	7.97 ± 3.74 ab (337.23 ± 141.46) *N* = 6	−0.63 ± 0.26 a (−69.05 ± 22) *N* = 8	−6.5 ± 1.71 ab (−70.83 ± 7.17) *N* = 11	4.05 ± 2.29 ab (175.18 ± 59.89) *N* = 8
OA Towel	10.22 ± 0.99 ab (883.25 ± 316.75) *N* = 2	1.38 ± 1.34 b (156.04 ± 90.11) *N* = 12	−5.72 ± 2.27 ab (11.68 ± 32.49) *N* = 12	−1.51 ± 2.47 ab (−2.12 ± 50.94) *N* = 6

Starting *V. destructor* populations influenced how well a treatment would reduce a mite population, with higher starting populations leading to lower treatment impact for treatments overall in winter (*t* = -5.08, *P* < 0.001; Supplementary Fig. S3A), spring (*t* = −16.4, *P* < 0.001; Supplementary Fig. S3B), and summer (*t* = −5.92, *P *< 0.001; Supplementary Fig. S3C). This is also true for the following specific treatments across all seasons: Apistan (*F*_3,32_ = 10.48, *P* < 0.001; Supplementary Fig. S3D), Bovitraz (*F*_3,25_ = 69.79, *P *< 0.001; Supplementary Fig. S3E), OA Dribble (*F*_3,28_ = 14.46, *P* < 0.001; Supplementary Fig. S3F), MAQS (*F*_3,24_ = 156.14, *P *< 0.001; Supplementary Fig. S3G), and OA towels (*F*_3,28_ = 13.02, *P* < 0.001; Supplementary Fig. S3H). Finally, there were significant interactions between season and initial *V. destructor* infestation rates for: Apiguard (*F*_3,26_ = 17.95, *P* < 0.001; Supplementary Fig. S4A), and CheckMite+ (*F*_3,25_ = 4.21, *P *= 0.015; Supplementary Fig. S4B).

### Experiment 2—Seasonal Efficacy

#### Fall efficacy trial.

**Fig. 3.**
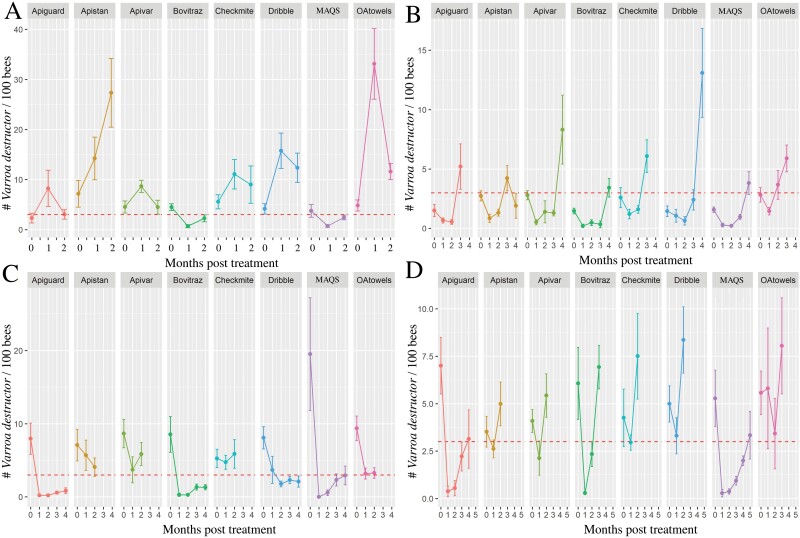
Experiment 2—*Varroa destructor* infestation rates (number of mites/100 adult bees = *y*-axis) for each miticide by seasonal trial. A) Fall, B) Winter, C) Spring, and D) Summer. The points represent average infestation rates, while the whiskers indicate standard errors. The dashed line indicates the standard economic threshold of 3 mites/100 bees. OA, oxalic acid.

Only 64.5% of the 96 experimental colonies used in this trial (12 colonies per treatment group) survived throughout the experimental period. Colonies in the CheckMite+ (42% survival) and the OA shop towel (17% survival) treatments had especially low survival rates (Fig. 2A). However, we experienced some unexpected environmental issues (high ant infestations and flooding in low areas) that likely impacted the survivorship in these 2 treatment groups, making it difficult to attribute the colony losses to high *V. destructor* infestations. *Varroa destructor* population resurgence in colonies after treatment with a miticide is shown in Fig. 3A. All the miticides failed to reduce *V. destructor* infestation rates below those of the baseline established at the beginning of the experiment, except for the Bovitraz and MAQS treatments, which were nearly at the 3 mites/100 bee threshold 2 months after treatment (Fig. 3A).

#### Winter efficacy trial.

The winter trial included 128 colonies, with 16 colonies per treatment group, with 77% of colonies surviving throughout the experimental period (Fig. 2B). All treatments applied during winter reduced mite populations, with the exception of the OA shop towel treatment (Table 2; Fig. 3B). *Varroa destructor* population reached the economic threshold of 3 mites/100 bees 3 months posttreatment for the Apiguard, Apistan, CheckMite+. Only the Apivar, Bovitraz, and MAQS treatments lasted 4 months posttreatment before reaching the 3 mites/100 bee threshold.

#### Spring efficacy trial.

The spring trial included 103 colonies (*n* = 13 colonies for each treatment group except for the MAQS group, for which *n* = 12). Most colonies (i.e., over 70%) survived during the experimental period within the spring trial (Fig. 2C); however, colonies treated with MAQS only had a 33% survival rate.

Like the winter trial, all treatments at least reduced *V. destructor* populations for 1 month (Fig. 3C), though the reduction was minor for both the Apistan, Apivar, and CheckMite+ treatments. Apiguard, Bovitraz, and the MAQS treatments reduced significantly more *V. destructor* during this spring trial than the Apistan and CheckMite+ treatments (Table 2). *Varroa destructor* population resurgence in colonies after treatment with a miticide is shown in Fig. 3C.

#### Summer efficacy trial.

There were 85 colonies used in this trial, with the Apiguard, CheckMite+, and Bovitraz treatments having 10 colonies per treatment and the Apistan, OA dribble, MAQS, OA towels, Apivar treatments having 11 colonies each. Colony survival during the experimental period was only 62% within the summer trial (Fig. 2D). In this trial, the MAQS- and Apiguard-treated colonies had the lowest survival rates, at 33% and 50%, respectively.

All treatments initially reduced *V. destructor* populations for 1 month except the OA shop towel treatment (Fig. 3D). *Varroa destructor* populations in colonies in 4 treatment groups (Apivar, Apistan, CheckMite+, and OA dribble) exceeded pretreatment levels 2 months after treatment application (Table 2). *Varroa destructor* population resurgence in colonies was slowest after the Bovitraz treatment, reaching 3 mites/100 bees after 2 months posttreatment. Both Apiguard and MAQS treatments had a slower population resurgence, but many colonies perished during the treatment period (Fig. 3D).

## Discussion

Our primary goal with these experiments was to measure the *V. destructor* population resurgence rates after chemical treatments. We evaluated 9 chemical treatments on 472 colonies to understand the relationship between *V. destructor* growth, season, and several popular chemical options currently available to US beekeepers. Our data support anecdotal reports from commercial beekeepers who state that they must treat colonies at least 4 times per year to keep *V. destructor* populations at manageable levels. Unfortunately, our data also highlight the ineffectiveness of some commercial products at keeping *V. destructor* populations low and accentuate the need for beekeepers to have new tools that are more effective controls for this mite. Thus, we believe our results can lead to better timing of treatment applications and reduce the overreliance on a single treatment type, thus slowing the expansion of *V. destructor* resistance while more effective treatment options are discovered and developed.

While many have reported *V. destructor* resistance to *tau*-fluvalinate in the United States (Baxter et al. 1998, Pettis et al. 1998, Elzen and Westervelt 2004), our results show that Apistan can still be used intermittently against the mite. We observed that Apistan at least reduced *V. destructor* populations for 1 month during the fall of 2014, although no reduction was observed during the fall of 2020. However, when applying Apistan during the winter, *V. destructor* population resurgence was delayed for 2 months. While these findings show that *tau*-fluvalinate resistance is certainly still present in Florida, Apistan may be a product beekeepers can use rotationally during times of the year when *V. destructor* populations are elevated, and colonies are broodless.

Amitraz is the synthetic active ingredient most used by beekeepers to control *V. destructor* in the United States (Haber et al. 2019). However, resistance to amitraz is being observed all over the country (Rinkevich 2020). Interestingly, in 2014, Apivar (3.3% amitraz) delayed *V. destructor* population resurgence for 2 months posttreatment in the fall season. Later in 2021, we observed that Apivar delayed *V. destructor* resurgence for 2 months in the winter but only delayed resurgence for 1 month in the other seasons.

The Bovitraz treatment, which has a higher concentration of amitraz than Apivar (6.25% amitraz), was the only synthetic miticide tested that delayed *V. destructor* population resurgence in each season, though its effect was still greatly reduced in the summer and fall seasons. Thus, a higher percentage of amitraz may be effective at reducing *V. destructor* populations. The amount of amitraz applied to colonies via the use of Bovitraz can possibly be reached in a hive simply by applying more Apivar strips if such an application is consistent with label directions. This could bring Apivar efficacy up to the same level as that seen in Bovitraz-treated colonies. However, increasing the amount of amitraz put into the colony could risk bee health. Therefore, research related to the amount of amitraz that builds up in the hive during treatment is important. The recently released Amiflex treatment by Veto-Pharma includes 2% amitraz, but it is a short-term “flash” treatment that may be applied quickly between honey flows or other treatments. We caution that continual usage of amitraz treatments, labeled or not, may only exacerbate resistance issues if other active ingredients are not utilized. Additionally, we feel it necessary to reemphasize that using off-label treatments is illegal and potentially dangerous to both human and environmental health.

There is a high level of resistance of *V. destructor* in Florida to coumaphos, the active ingredient in CheckMite+. We observed that CheckMite+ failed to reduce *V. destructor* populations in the fall and only reduced populations for a single month in the winter, spring, and summer seasons. Our studies add to the growing body of research showing widespread mite resistance to this compound (Spreafico et al. 2001, Elzen and Westervelt 2002, Pettis 2004, Maggi et al. 2009).

Results from both natural volatile compounds tested (thymol in Apiguard and formic acid in Mite Away II and MAQS) provided evidence that these compounds can be utilized by beekeepers to reduce *V. destructor* populations effectively. We observed that Apiguard significantly delayed *V. destructor* resurgence in the winter and spring seasons in Florida, but it was not significantly more effective than other treatments during summer and fall. Although Apiguard reduced *V. destructor* populations for 2 months during the summer, many colonies treated with Apiguard perished. Many beekeepers use thymol-based products worldwide (Jack and Ellis 2021), as these treatments have been shown by many to reduce *V. destructor* populations by 50%–80% (Melathopoulos and Gates 2003, Gregorc and Planinc 2012, Coffey and Breen 2013). However, it is well known that thymol can be harmful to honey bee broods when applied during periods of high ambient temperatures (Calderone 1999, Floris et al. 2004).

Colonies treated with formic acid via the product Mite Away II experienced the greatest delay in *V. destructor* population resurgence in the fall of 2014. The other formic acid treatment tested, MAQS, significantly delayed *V. destructor* populations for 2 months posttreatment in the winter, spring, and summer seasons, though we only observed 1 month of delayed resurgence in the fall. This long-lasting success may have been due to formic acid’s ability to affect mites that are inside of the brood cells (Fries et al. 1991), delaying the *V. destructor* reproduction during treatment. Treatments with formic acid as their main active ingredient are especially known for being harsh on honey bee queens (Underwood and Currie 2004, 2005). When using these treatments appropriately, we did not observe any issues with queen loss in either of our experiments. However, we do not recommend using a formic acid treatment during periods when the average ambient temperature is beyond the labeled temperature use range, as it can impact colony health negatively, as stated on the label. Nevertheless, newer formulations of formic acid products have wider temperature windows; thus, we recommend that beekeepers follow the label concerning temperature recommendations in their area.

Of the 2 OA treatments we tested, the OA dribble method significantly delayed *V. destructor* resurgence longer in the winter than the OA shop towel method, but not during the other seasons. The dribble method is a labeled application of OA in the United States and has been shown by several others to be an effective method for controlling *V. destructor* populations (Gregorc et al. 2016, 2017, Kulhanek et al. 2022). As OA cannot penetrate the brood cappings (Gregorc and Planinc 2001), the OA dribble method is most effective when colonies have less brood or are completely broodless. The shop towel method is not a labeled application of OA. Yet, many US beekeepers are currently interested in an extended-release OA method, and many homemade recipes exist online. Unfortunately, the method we used was incredibly ineffective in the fall and winter and only moderately effective for 2 months in the spring and summer seasons. At best, this extended-release OA method could be utilized to limit *V. destructor* population growth but not to reduce mite populations. The use of OA via the product Api-Bioxal is allowed during periods when honey supers are present. Thus, OA can be considered a valuable tool when used as part of an integrated approach to *V. destructor* control, especially during periods in which there is little or no brood present in the colony. Ultimately, developing a method for improved extended release of OA should remain a research priority. We did not test OA vaporization (another labeled application method for OA). This method of OA application has been shown by others to be the most effective OA application method to reduce *V. destructor* populations (Al Toufailia et al. 2015).

There are other natural products, such as HopGuard III and Formic Pro, on the market for US beekeepers, and these were not tested in the current study. They, too, should be evaluated seasonally. Additionally, other researchers have shown that beekeepers in the United States will treat colonies with multiple compounds simultaneously, for example, by combining natural compounds with synthetic miticides such as amitraz (Haber et al. 2019). Aurell et al. (2023) recently explored the combination of amitraz and thymol and found an increased efficacy when combining the 2 treatments during fall. Thus, co-treatment with multiple miticides could be explored as a possible way to improve *V. destructor* control, but only if treatment with multiple compounds is allowed on the product labels.

During Experiment 2, we observed extremely high colony mortality within the 3-month experimental period during our fall trial. To compare the survivorship of colonies across seasonal treatments, we limited our survival analysis to a 3-month period instead of following the colonies for several months after treatment. This, unfortunately, prevents us from truly observing the impacts of *V. destructor* on honey bee colony survival in this experiment and rather only provides us with information regarding seasonal treatment on colony survival. Fortunately, the work of Jack et al. (2023) specifically demonstrates the impact of seasonal *V. destructor* population growth on honey bee colony health over many months following intensive mite treatments. They found that about 4–5 months after treatment in the winter and spring months *V. destructor* populations would return to the standard economic threshold of 3 mites/100 bees, putting colonies at greater risk of collapse 4–6 months after crossing the threshold. In the summer and fall months, mite populations would cross the economic threshold only 2–3 months after treatment, again leading to collapse a few months later. Thus, beekeepers should ensure they are applying effective treatments during the winter and spring months to preserve their colonies.

During the winter, beekeepers have many options to control *V. destructor* populations, as all treatments tested reduced mite populations except for CheckMite+ and the OA shop towels methods. In the spring, there are still several effective treatment options, such as Apiguard, MAQS, OA dribble, and Bovitraz delayed *V. destructor* resurgence. However, in the summer, treatment options are more limited, as only MAQS significantly reduced *V. destructor* populations, while Apiguard, OA shop towels, and Bovitraz barely delayed *V. destructor* resurgence during the season. During the fall, we only delayed *V. destructor* resurgence using formic acid and Bovitraz treatments. Unfortunately, the efficacy and safety of formic acid depend heavily on the ambient temperature, making it an unsuitable treatment for many beekeepers during the summer and fall seasons. Furthermore, Bovitraz is an off-label treatment that only reduced *V. destructor* by ~14%–40% 2 months posttreatment during these periods, and it is not legally permitted to be used by beekeepers due to environmental and human health concerns. To improve mite control in summer and fall, it is important to continue searching for new active ingredients that can be registered for use against *V. destructor* or modify application methods of existing treatments (Dulin et al. 2014, Riva et al. 2019, Bahreini et al. 2020, Jack et al. 2020, 2022). Another option is to improve formulations of existing active ingredients, especially amitraz, to increase their efficacy. The amitraz-based Amiflex product, recently registered in the United States, is an example of the importance of formulating products to fit specific situations. Ultimately, beekeepers should adopt an integrated pest management strategy employing multiple nonchemical techniques and using chemical treatments as necessary, always in rotation (Jack and Ellis 2021).

As beekeepers struggle to control *V. destructor* globally, it is essential that beekeepers optimize the treatments currently available to them while waiting for new treatments to be developed. We believe that the research described herein will be of great value to beekeepers in Florida and elsewhere. While mite infestation levels and treatment efficacies may be region-specific, our work demonstrates the importance of frequent *V. destructor* monitoring, even after treatment. Many beekeepers treat their honey bee colonies on a calendar schedule and never confirm through sampling if their treatments sufficiently reduce *V. destructor* populations. Additionally, we identified clear seasonal variations in treatment efficacy, once again underscoring the need for beekeeper monitoring. Even if a beekeeper has a satisfactory reduction of *V. destructor* populations in a honey bee colony with 1 treatment, it does not mean they will get the same level of reduction with a repeat application later in the year. Future research should focus on repeating this seasonal efficacy of miticide work in different regions throughout the United States to provide beekeepers with specific treatment efficacy and seasonality information related to where they keep bees. Furthermore, there are many other chemical and nonchemical treatments we did not test, treatments that would be interesting to include in future comparisons. This research was an important step forward toward optimal usage of current chemical treatments for *V. destructor* control.

## Supplementary Material

ieae011_suppl_Supplementary_Figures_1-4

ieae011_suppl_Supplementary_Tables_1
