# Supplementary material for: Evaluating the seasonal efficacy of commonly used chemical treatments on Varroa destructor (Mesostigmata: Varroidae) population resurgence in honey bee colonies
Source: J Insect Sci. 2024 May 28;24(3):11. doi: 10.1093/jisesa/ieae011 (PMC11132127; doi:10.1093/jisesa/ieae011)
Supplement: ieae011_suppl_Supplementary_Figures_1-4 [file ieae011_suppl_supplementary_figures_1-4.zip › Supplementary_Figure_Captions.docx]

**Supplementary Figure 1.** Experiment 1 – Proportion of experimental honey bee colonies that survived each experimental month. Treatment did not affect the proportion of colonies that survived (*P* = 0.505).

**Supplementary Figure 2.** Experiment 1 – The difference in *Varroa destructor* infestation rates between immediately post treatment and pre-treatment plotted against pre-treatment *V. destructor* infestation rates (# mites/100 adult honey bees). A: These two parameters are not significantly correlated, meaning that overall treatment efficacy was unaffected by starting mite infestation rates. B: There was a significant interaction between the pre-treatment *Varroa* infestation rates and the difference between pre- and post-treatment *Varroa* infestation rates, meaning that the directionality of the effect for CheckMite+® was significantly different from the effect of other treatments. Output represents estimated marginal means based on the linear model.

**Supplementary Figure 3:** Experiment 2 – Treatment efficacy plotted against pre-treatment *Varroa destructor* infestation rates (# mites/100 adult honey bees). These two parameters are negatively correlated, meaning that treatment efficacy improved at lower starting mite infestation rates. All graphs are based on the estimated marginal means of the linear model. Only the cases in which the negative correlation between seasons and treatments were significant are shown. (A) Overall winter season. (B) Overall spring season. (C) Overall summer season. (D) Apistan®. (E) Bovitraz®. (F) Oxalic acid dribble. (G) MAQS®. (H) Oxalic acid towels.

**Supplementary Figure 4.** Experiment 2 – Treatment efficacy plotted against pretreatment *Varroa destructor* infestation rates (# mites/100 adult honey bees). These plots represent the two parameter interactions between pre-treatment *V. destructor* infestation and seasons for the cases shown. This means that treatment efficacy improved at lower starting mite infestation rates in some seasons and not in others. All graphs are based on the estimated marginal means of the linear model. (A) Apiguard®. (B) CheckMite+®.
