# Supplementary figures and images for: Evaluating the seasonal efficacy of commonly used chemical treatments on Varroa destructor (Mesostigmata: Varroidae) population resurgence in honey bee colonies
Source: J Insect Sci. 2024 May 28;24(3):11. doi: 10.1093/jisesa/ieae011 (PMC11132127; doi:10.1093/jisesa/ieae011)

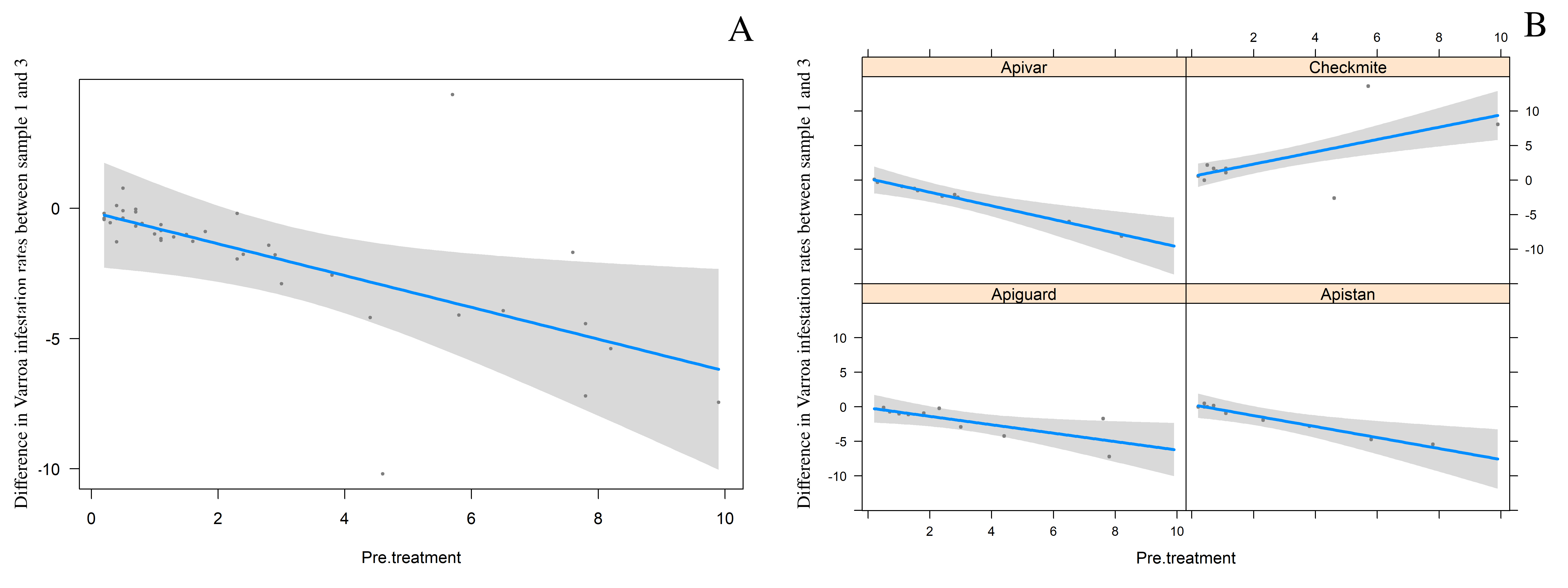

Supplement: ieae011_suppl_Supplementary_Figures_1-4 [file ieae011_suppl_supplementary_figures_1-4.zip › ExptS1_FigS2.png]

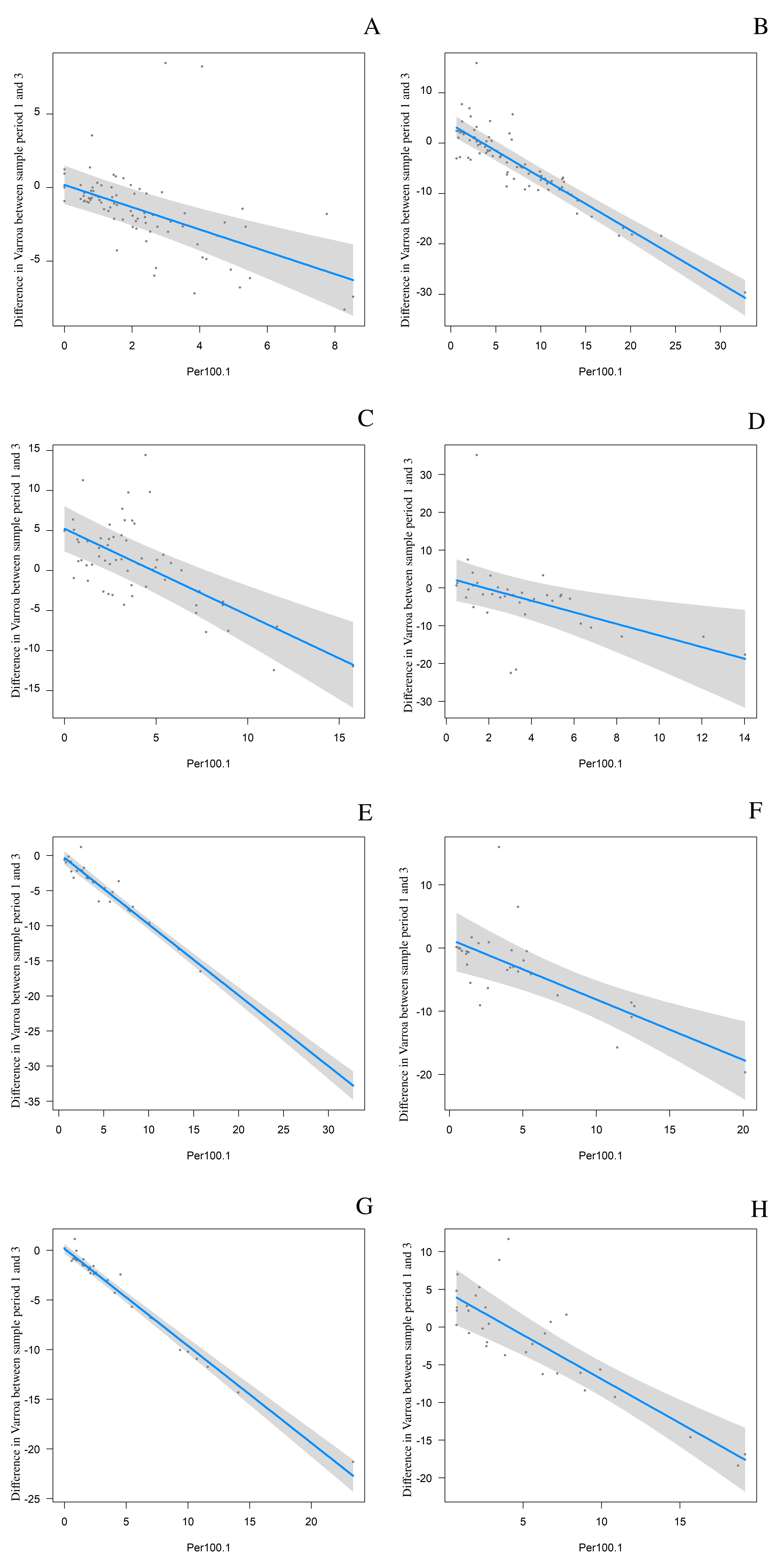

Supplement: ieae011_suppl_Supplementary_Figures_1-4 [file ieae011_suppl_supplementary_figures_1-4.zip › ExptS2_FigS3.png]

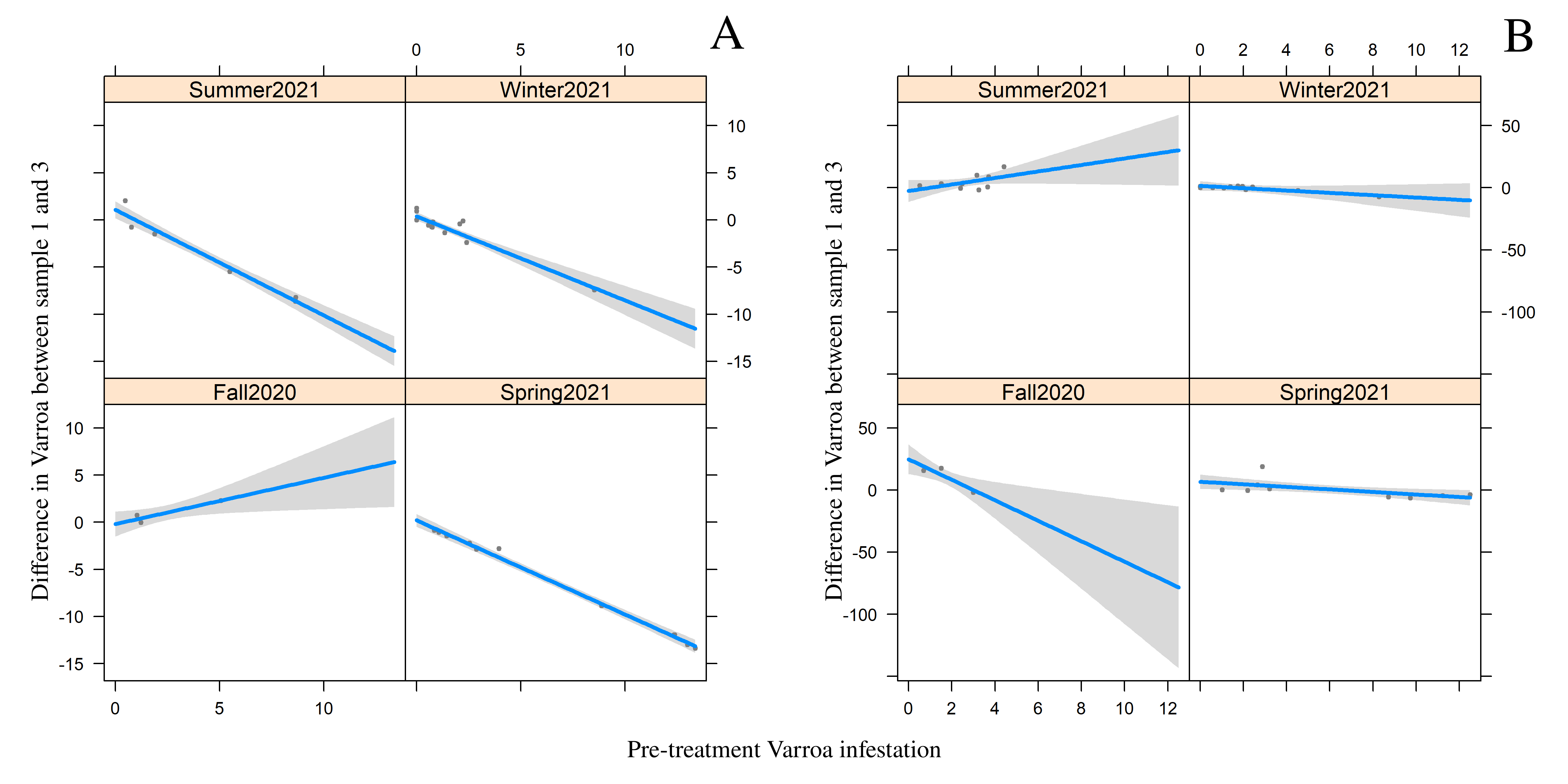

Supplement: ieae011_suppl_Supplementary_Figures_1-4 [file ieae011_suppl_supplementary_figures_1-4.zip › ExptS2_FigS4.png]

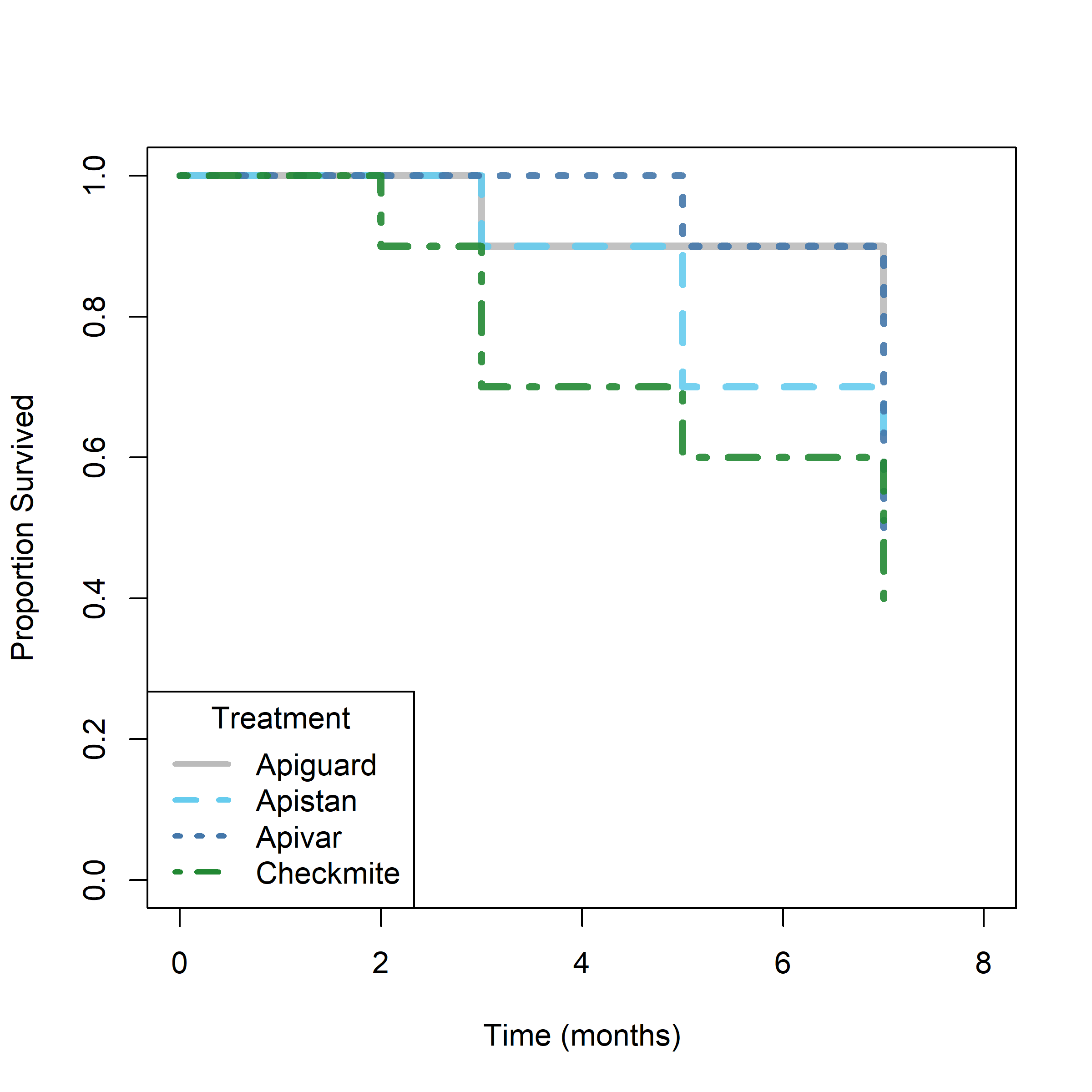

Supplement: ieae011_suppl_Supplementary_Figures_1-4 [file ieae011_suppl_supplementary_figures_1-4.zip › ResurgenceSurvival_FigS1.png]
