# Supplementary material for: Evaluating the seasonal efficacy of commonly used chemical treatments on Varroa destructor (Mesostigmata: Varroidae) population resurgence in honey bee colonies
Source: J Insect Sci. 2024 May 28;24(3):11. doi: 10.1093/jisesa/ieae011 (PMC11132127; doi:10.1093/jisesa/ieae011)
Supplement: ieae011_suppl_Supplementary_Tables_1 [file ieae011_suppl_supplementary_tables_1.docx]

**Supplementary Table 1.** *Experiment 1* Average difference in *Varroa destructor* infestation rates between pretreatment and immediately post treatment (September – October 2014) for each season, defined as treatment efficacy. Values in parentheses are the percent change in *V. destructor* infestation rates during the treatment period. The data are mean ± s.e. Negative numbers represent a decrease in *V. destructor* infestation rates over the designated treatment period while positive numbers represent an increase in infestation rates. Means with different letters are significantly different at α ≤ 0.05.

| Treatment | Fall 2014 |
| --- | --- |
| Apiguard**®** | -2 ± 0.7 (-0.67 ± 0.12) a  N=10 |
| Apistan® | -1.5 ± 0.68 (-0.23 ± 0.21) a  N=10 |
| Apivar® | -2.5 ± 0.82 (-0.75 ± 0.14) a  N=10 |
| CheckMite**+®** | 2.8 ± 1.47 (1.66 ± 0.46) b  N=10 |
